# Enhanced Cell Adhesion on a Nano-Embossed, Sticky Surface Prepared by the Printing of a DOPA-Bolaamphiphile Assembly Ink

**DOI:** 10.1038/s41598-017-14249-4

**Published:** 2017-10-23

**Authors:** Chaemyeong Lee, Seung-Hyun Kim, Jae-Hyung Jang, Sang-Yup Lee

**Affiliations:** Department of Chemical and Biomolecular Engineering, Yonsei University, 50 Yonsei-ro, Seodaemun-gu, Seoul, 120–749 Korea

## Abstract

Inspired by adhesive mussel proteins, nanospherical self-assemblies were prepared from bolaamphiphiles containing 3,4-dihydroxyphenylalanine (DOPA) moieties, and a suspension of the bolaamphiphile assemblies was used for the preparation of a patterned surface that enhanced cell adhesion and viability. The abundant surface-exposed catechol groups on the robust bolaamphiphile self-assemblies were responsible for their outstanding adhesivity to various surfaces and showed purely elastic mechanical behaviour in response to tensile stress. Compared to other polydopamine coatings, the spherical DOPA-bolaamphiphile assemblies were coated uniformly and densely on the surface, yielding a nano-embossed surface. Cell culture tests on the surface modified by DOPA-bolaamphiphiles also showed enhanced cellular adhesivity and increased viability compared to surfaces decorated with other catecholic compounds. Furthermore, the guided growth of a cell line was demonstrated on the patterned surface, which was prepared by inkjet printing using a suspension of the self-assembled particles as an ink. The self-assembly of DOPA-bolaamphiphiles shows that they are a promising adhesive, biocompatible material with the potential to modify various substances.

## Introduction

Surface modification to increase the adhesion and proliferation of cells is of interest in biological and biomedical research for the use of various synthetic materials such as fluorinated polymers^1^ and metals^2,3^. To date, the surfaces of these substances have been mostly modified by chemical methods or by the physical fabrication of micro/nano-scaled features^4–7^. For example, chemical modification methods include using silane coupling agents^8–10^, the adsorption of polyelectrolyte multilayers^11,12^, or coating with catechol-derivatives including polydopamine^13–15^. After chemical modification, many hydrophobic surfaces were transformed into hydrophilic surfaces to increase biocompatibility and water wettability^16,17^. On the other, the creation of micro/nanostructures on the substrate surface enhances the cell adhesivity by providing structural features of a suitable size for cell protein attachment^18^, although the optimal size is dependent on the cell characteristics^19,20^. Generally, a feature size around 100 nm is advantageous for cell adhesion, and the cell compartments interact with the surface features positively^21^.The combination of both chemical and physical methods has also been widely explored; for example, roughened surfaces with micro/nano features have been created by photolithography processing or photonic crystal assembly, followed by the structured surface being coated with chemicals to improve biological compatibility^22–24^. Previous studies have demonstrated that the combined use of both physical and chemical methods enhances cell adhesivity and proliferation. However, the two-step surface modification process is difficult and costly. In addition, the materials applicable for the manufacture of submicron features are limited. Therefore, a facile method for surface modification is required, where the formation of micro/nanostructures and an increase in the chemical affinity of the surface for biomolecules can be simultaneously achieved for a variety of substrate surfaces.

The amino acid abundant in the mussel foot protein, 3,4-dihydroxyphenylalanine (DOPA), shows outstanding adhesivity to various surfaces due to the catechol groups that can form hydrogen bonds or coordinate to metal compounds^25–27^. To exploit the biochemical properties of DOPA, we recently produced a DOPA-containing bolaamphiphilic molecule (DOPA-C7 hereafter) and demonstrated that its self-assembly was robust and stable even under vacuum and in dry conditions, where the adhesivity from the surface-exposed catechols was retained. Due to these physical and chemical properties, the DOPA-C7 assemblies could be applied as nanoscale spherical sticky templates for the preparation of magnetic nanoparticles^28^. Consequently, the DOPA-C7 assemblies are promising surface-modification agents, which impart adhesivity and rigidity.

**Figure 1:**
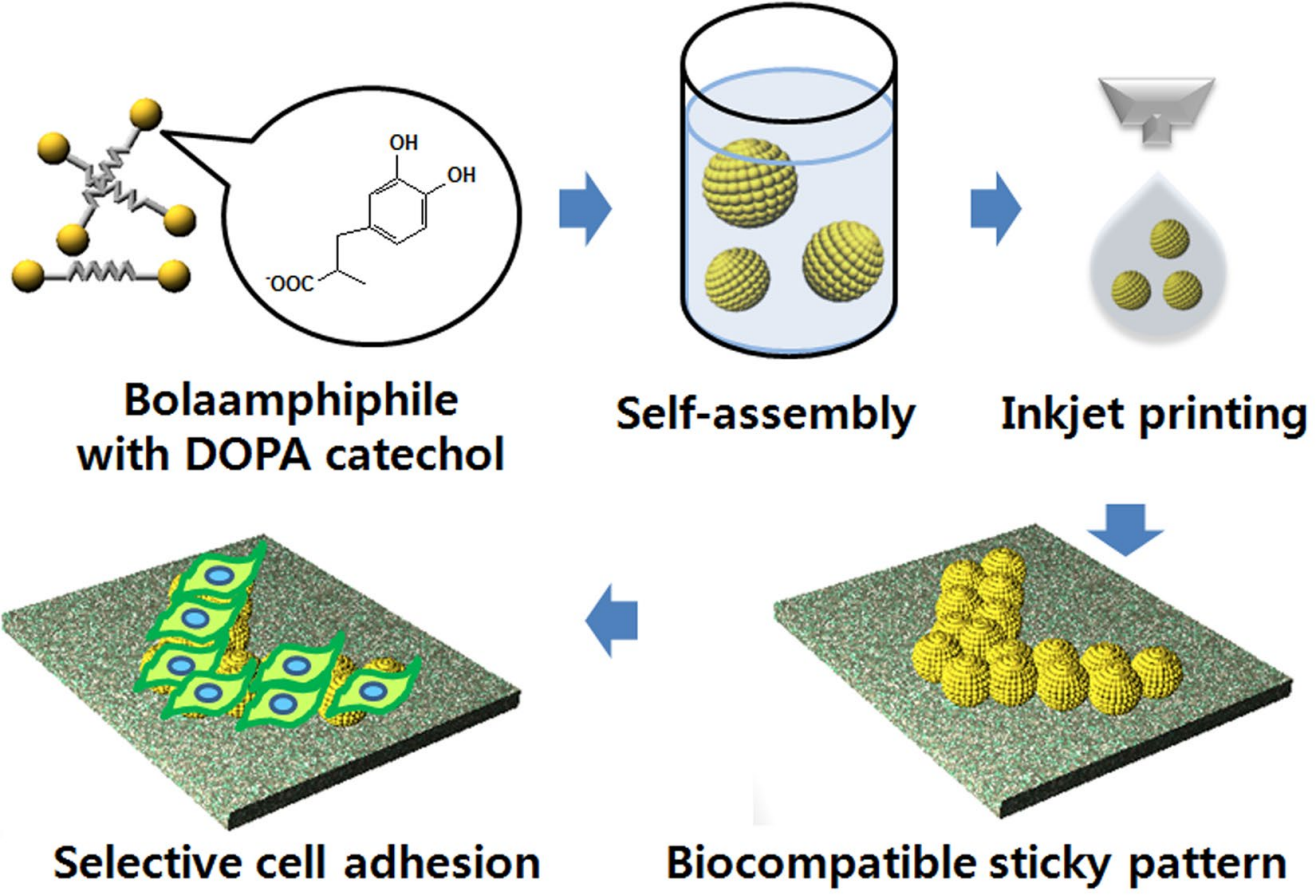
Schematic illustration of the selective cell adhesion on a patterned surface that was prepared by the inkjet printing of bolaamphiphile assemblies.

In this paper, a versatile route for surface modification using DOPA-C7 assemblies is demonstrated. The hydrophobic surface modified by the DOPA-C7 assemblies exhibited remarkably enhanced cell adhesivity and viability. Furthermore, the uniform deposition of spherical assemblies results in the creation of a nano-embossed surface. The adhesivity and nano-embossed surface features on the modified surface were characterized by macro/microscopic adhesion force tests and other surface analyses. For the modified surfaces, cell adhesion and viability were tested using the NIH-3T3 and PC12 cell lines. We also examined pattern printing on a polytetrafluoroethylene (PTFE) substrate with a commercialized inkjet printer using the DOPA-C7 assembly suspension as an ink for guided cell growth on the pattern (Fig. 1).

## Results

### Characterization of the modified surface

**Figure 2:**
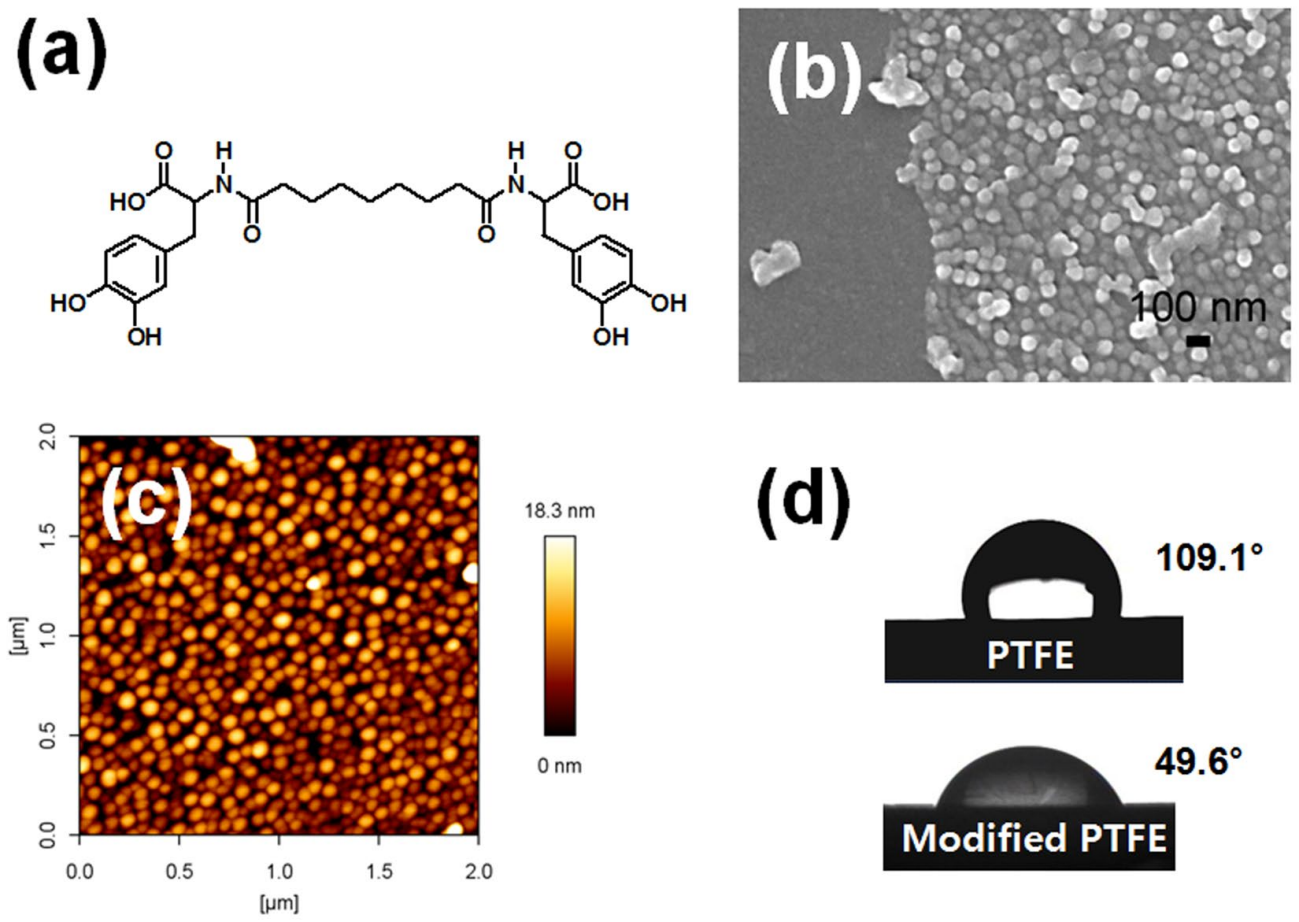
Surface modification using the bolaamphiphile assemblies. (**a**) Molecular structure of bis(*N*-alpha-amido-3,4-dihydroxyphenylalanine)−1,7-heptane dicarboxylate (DOPA-C7), (**b**) SEM image of the modified surface of silicon wafer (left smooth surface: bare silicone, right embossed surface: modified with DOPA-C7 assemblies), (**c**) topological image of the modified surface showing densely deposited assemblies, (**d**) photo images of the contact angle before/after surface modification of PTFE film. The hydrophobic PTFE surface (109.1°) became hydrophilic (49.6°) after the modification.

The DOPA-C7 molecule has a symmetric structure, composed of a central heptyl chain and two DOPA moieties at the ends (Fig. 2a). When DOPA-C7 molecules are dissolved in aqueous solutions, they spontaneously self-assemble, creating nanospherical structures with exposed DOPA catechol moieties on the surface. These nanospherical assembled structures are likely to be obtained by the aggregation of the DOPA-C7 and subsequent assembly of those aggregates^29^. Briefly, the DOPA-C7 molecules quickly aggregate to form small clusters in aqueous solution by the hydrophobic interaction between alkyl chains. These clusters were assembled further through the hydrogen bond working between the dihydroxyphenyl groups to form spherical assembled structures. The prepared assemblies were 87.5 ± 9.1 nm in diameter and maintained their spherical shapes under dry conditions without collapse. This DOPA-C7 assembly suspension was applied as an adhesive to modify the surfaces of various substrates. To demonstrate the adhesivity, we stamped the DOPA-C7 assemblies on a silicon wafer using a PDMS slab as a stamp and the DOPA-C7 suspension as ink. SEM images of the stamped surface showed that the spherical DOPA-C7 assemblies were deposited densely with clear visualization of each assembled particle (Fig. 2b). The clear boundary of the stamped layer indicates that DOPA-C7 assembly suspension is applicable for surface modification with a fine feature size. The DOPA-C7 assembly particles remained unchanged, leading to the formation of a uniform coating on the surface (see Supplementary Information, Fig. S1a). Remarkably, the uniformity of the DOPA-C7 coated layer was better than that of a polydopamine (PDA) coating. When we tested PDA as a coating material using the same stamping method, the surface was not uniformly coated, and many large PDA aggregates and uncoated areas were visible (see SI, Fig. S1b). The non-uniform coating makes the application of PDA as a smooth, sticky coating on various substrates difficult^30–32^. The PDA aggregates likely form during the dopamine polymerization step. The detailed structure of the modified surface was observed further by atomic force microscopy (AFM) topology studies, and each DOPA-C7 assembly can be clearly seen (Fig. 2c). Furthermore, the height profile confirms the creation of a nano-embossed surface with a height of 10 nm after the deposition of the DOPA-C7 spherical assemblies (see SI, Fig. S2).

The changes in the water contact angle most clearly display the effect of the surface modification. Figure 2(d) shows changes in the water contact angle after deposition of the DOPA-C7 assemblies on the PTFE substrate. The average contact angle on PTFE was reduced from 109.1 ± 0.1° to 49.6 ± 2.5° (*n* = 3), indicating the successful transformation of the surface from hydrophobic to hydrophilic. This surface modification method was applicable for other substrates including polyethylene terephthalate (PET), silicon rubber, and glass (see SI, Fig. S3). The reduction in the water contact angle is mainly due to the catechol moieties on the surface of the DOPA-C7 assemblies rather than the nano-embossed features because their scale is too small to influence the water contact angle.

**Figure 3:**
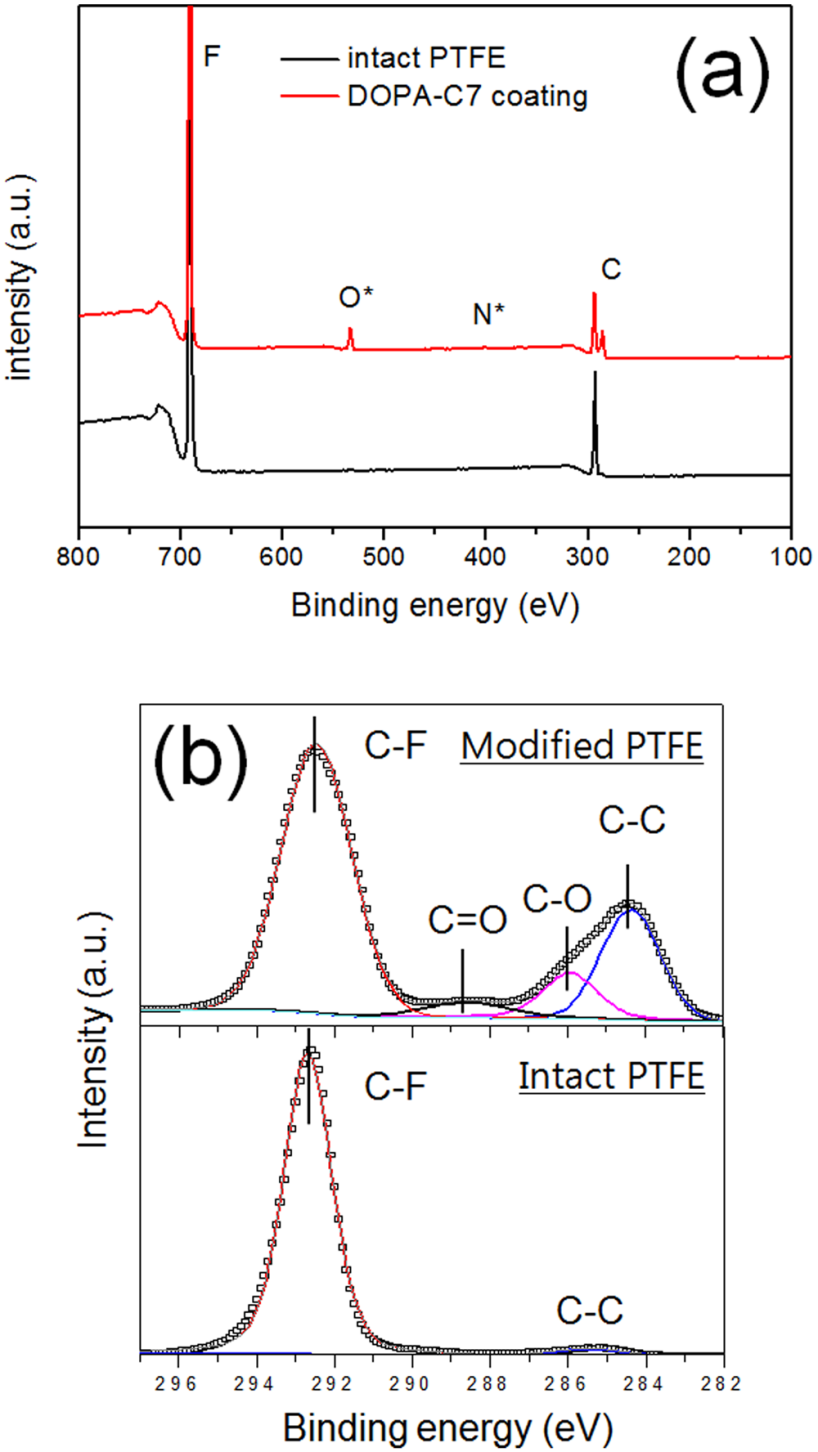
XPS spectra of the modified surface. (**a**) XPS survey spectra of the untreated and modified PTFE surfaces. (**b**) Deconvoluted C1s spectra showing the appearance of C-O and C = O bonds from the coating layer.

The modified surface was further analysed using X-ray photoelectron spectroscopy (XPS). The XPS spectra of the PTFE surfaces before/after DOPA-C7 modification are shown in Fig. 3. The unmodified PTFE surface showed only F1s and C1s peaks. After the deposition of the DOPA-C7 assemblies, an O1s peak and weak trace of an N1s peak appeared (Fig. 3a). These peaks indicate the presence of DOPA-C7 on the PTFE surface, in which the oxygen peak originates from the hydroxyl catechol groups. A detailed analysis of the deconvoluted C1s peaks clearly revealed the differences in the surface species after the surface modification (Fig. 3b). Intact PTFE surfaces exhibited a strong peak corresponding to C-F (292.3 eV) bonds with a weak, broad shoulder corresponding to C-C (285.4 eV) bonds. In contrast, the modified PTFE displayed two characteristic peaks at 286.0 and 288.4 eV, corresponding to the C-O and C = O bonds of the quinone groups of catechol, respectively^33^. The intense C-C peak at 284.3 eV originates from the heptyl chain of DOPA-C7 rather than those of PTFE. Consequently, the XPS survey confirmed that the PTFE surface was covered with DOPA-C7 assemblies with exposed catechol hydroxyl groups.

**Figure 4:**
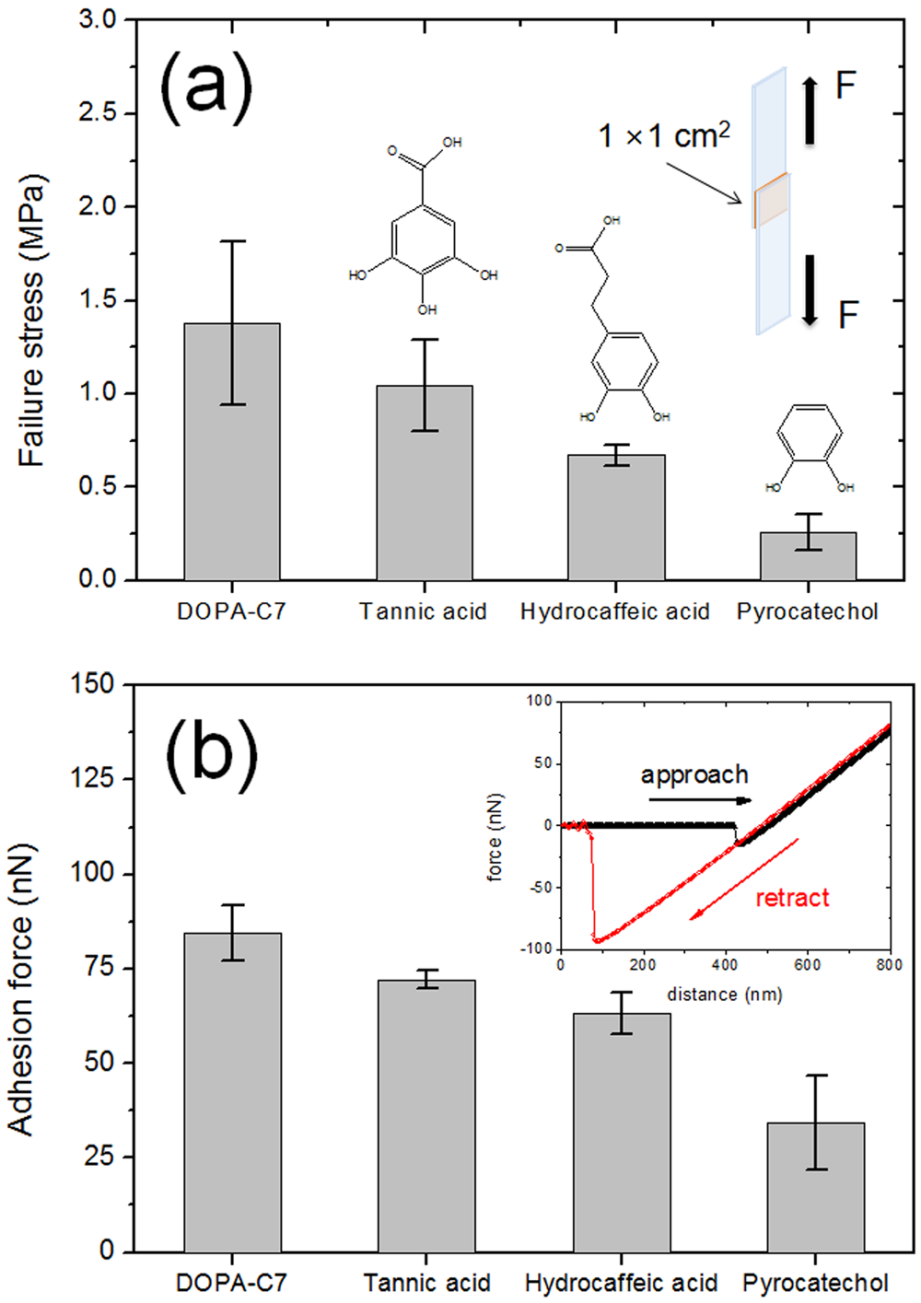
Determination of the adhesion strength of the DOPA-C7 assemblies at the macro- and microscopic level. (**a**) Failure stress of the DOPA-C7 assembly and other catechol compounds (*n* = 5). (**b**) The adhesion force of each compound was determined from the force-distance curve measured using AFM in contact mode. The inset figure shows a characteristic force-distance curve used for determination of adhesion force.

Aside from the embossed surface and enhanced water wettability, the DOPA-C7 assemblies show considerable adhesivity, which originates from the hydrogen bonding of catechol groups. First, we evaluated the adhesion strength of the DOPA-C7 assemblies at a macroscopic level. DOPA-C7 and other catechol compounds were applied as adhesives to bind two PET strips (Fig. 4a). The maximum stress at5 fracture of the binding was determined by applying pulling forces. DOPA-C7 had a failure stress of 1.38 ± 0.4 MPa, which is the highest among the catechol components examined. The high adhesion strength of DOPA-C7 might originate from the abundant catechol groups on the surface of the assemblies. Furthermore, the embossed surface may increase the surface area, thus increasing the binding strength. Notably, the assembled DOPA-C7 layer behaved like a solid, displaying almost a purely elastic stress-strain curve with a Young’s modulus of 1.64 MPa (see SI, Fig. S4). This purely elastic behaviour presumably resulted from the solid-like character of the DOPA-C7 assemblies. Although the spherical structure was produced by a self-assembly process, the assembled structures did not show viscous behaviour. This rigidity of DOPA-C7 assembly with high adhesivity is remarkable in that similar rigidity is observed from the solidified mussel foot protein. Consequently, the DOPA-C7 assemblies are suitable for the facile preparation of solid, adhesive layers on various surfaces.

The adhesivity of the DOPA-C7 self-assembly layer was further investigated at the microscopic level by AFM. The force–distance curve shows the adhesion strength of a surface at the submicron level. The inset of Fig. 4(b) shows a characteristic force-distance curve obtained from the approach and retraction steps of a Si_3_N_4_ probe on the modified surface. In agreement with the previous failure tests, DOPA-C7 showed the highest adhesion force, 84.6 ± 7.3 nN, compared to the other catechol-based compounds. This implies that more hydroxyl catechol groups are present than other catecholic compound layers in microscopic level. The AFM results confirmed that the enhanced adhesive property originates from the abundant catechol groups on the DOPA-C7 assemblies; in addition, the nano-embossing might increase the interaction area, allowing the formation of more hydrogen bonds with the AFM tip.

### Adhesion and viability of cells on the modified surfaces

A surface coating of catecholic compounds has been reported to enhance cell adhesion, although obtaining a uniform coating is still challenging^34,35^. Because the surfaces decorated by DOPA-C7 assemblies showed adhesivity, coating uniformity, and surface embossing at the nanoscale, we examined the cell adhesion and proliferation on the modified surface. For the clear demonstration of a sticky biointerface construction by DOPA-C7 assemblies, PTFE was chosen as a model substrate because of its low surface energy and low cell adhesivity^36,37^. Cellular behaviors on commercially available tissue culture plates (TCP) were monitored in parallel as a criterion to determine whether the adhesion and viabilities of cells on the DOPA-C7-treated substrates were comparably promoted as those on a normal cell culture condition.

**Figure 5:**
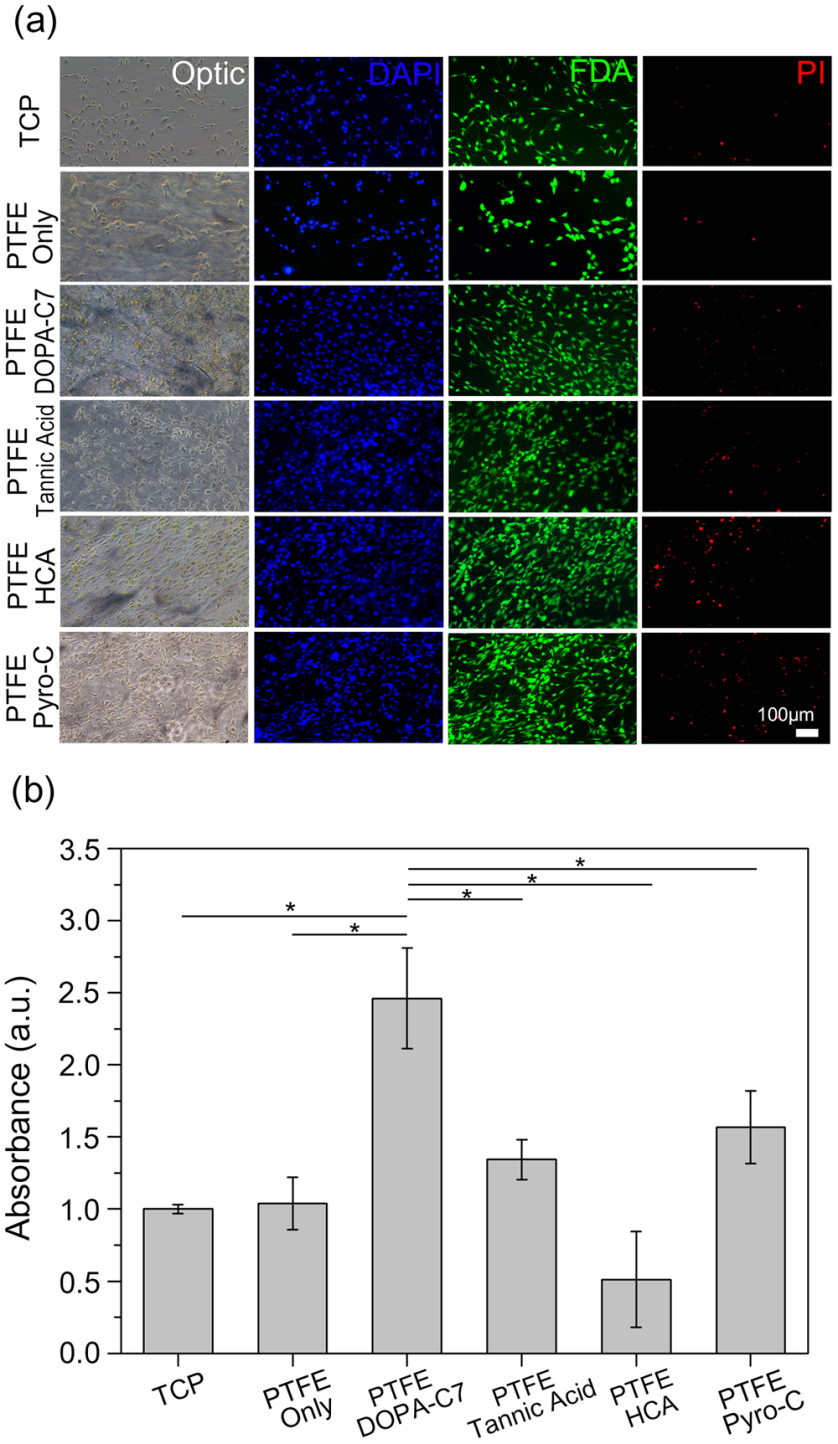
Adhesion test and live/dead assay of NIH-3T3 cells on the surface modified PTFE substrates. (**a**) Optical and fluorescence microscopy images representing the adhesivity and live/dead assay of the NIH-3T3 cell line stained with DAPI(blue), FDA(green), and PI(red) reagents (scale bar: 100 μm). (**b**) Relative viability of NIH-3T3 cell on the PTFE surfaces coated with DOPA-C7 assemblies. Relative viability of TCP: 1.00 ± 0.03; uncoated PTFE: 1.04 ± 0.18; DOPA-C7 coated PTFE: 2.46 ± 0.35; tannic acid coated PTFE: 1.34 ± 0.14; hydrocaffeic acid coated PTFE: 0.51 ± 0.33; and pyrocatechol coated PTFE: 1.57 ± 0.25 (p < 0.05).

As expected, cell adhesion was enhanced remarkably after the DOPA-C7 surface modification. Figure 5(a) shows the optical and fluorescence microscopy images of NIH-3T3 cells stained with 4′,6-diamidino-2-phenylindole/fluorescein diacetate/propidium iodide (DAPI/FDA/PI) reagents to indicate cell adhesion and the live/dead cell distribution on the modified substrates. Each substrate was rinsed with PBS prior to acquiring fluorescence images to evaluate the adhesive capabilities of each substrate. Consequently, the fluorescence microscopy images of FDA-stained cells demonstrate the significant improvement in the adhesion of live cells on the PTFE surfaces modified with DOPA-C7 or other catecholic compounds. Approximately three-fold increases in the number of FDA-stained cells were adhered on the substrates treated with DOPA-C7, hydrocaffeic acid, tannic acid or pyrocatechol compared to that obtained on the non-treated PTFE surface, further confirming the superiority of DOPA-C7 surface modification for promoting cell adhesion (left panel of Fig. S6).

The cell viability on the modified surface was quantitatively analysed using the cell counting kit 8 (CCK-8) assay^38^. The deviations in the cellular viabilities obtained by the CCK-8 assay, which are highly related to the number of live and dead cells adhered on each designated substrate, can present the extent of cellular adhesion at the surface with different adhesive properties^39,40^. The UV-vis absorbance intensity per unit area (λ = 450 nm) of each surface was normalized to the value obtained for the TCP surface. At 2 days post-culture, the DOPA-C7 assembly-decorated PTFE surface demonstrated significantly enhanced cellular viability compared to those on the other surfaces (Fig. 5b). The improved viabilities of cells on the PTFE/DOPA-C7 at 2 days post-culture can be attributed to the large number of live cells yet small number of dead cells on its surface compared to the other substrates (Fig. S6). The PTFE surface that was treated with HCA or tannic acid contained large number of live cells, but large numbers of dead cells also existed on the surface compared to those on the other substrate sets (Fig. S6), possibly reducing the cellular viabilities on each substrate (Fig. 5b) as discussed in previous studies^41,42^. Importantly, the improved cellular viability on the DOPA-C7 surface compared to that on the unmodified PTFE surface was further maintained at 4 days post-culture (Fig. S7). Cellular viabilities at early stage (2 days), possibly representing the levels of cellular adhesion and proliferation, were primarily improved on the PTFE/DOPA-C7 substrate (Fig. 5b), but no significant differences in cellular viabilities on its surface were observed at later stage (i.e., 4 days) compared to those on the TCP (Fig. S7), confirming its sufficient qualification as a cell culture substrate.

This higher cell absorbance intensity is indicative of the enhanced viability and lower toxicity on the DOPA-C7 assembly surface compared to the other catechol-modified surfaces. This low toxicity of the DOPA-C7 assembly is notable, especially considering that such low toxicity has been observed only in the polymerized dopamine (polydopamine) that precludes release of dopamine monomer^34^. The solid assembly presumably prevents the release of the DOPA-C7 monomers and displays a similar low-toxicity to that obtained after polymerization.

Generally, cell adhesion is thought to be initiated by the adhesion of proteins or glycoproteins secreted from a cell onto the surface of the substrate^43^. The adhesion of such biological components is achieved by means of multiple molecular interactions such as dispersion force, electrostatic attraction, hydrogen bonds, and hydrophobic interactions^44,45^. The deposition of the DOPA-C7 assembly on the substrate surely enhanced the hydrogen bonding between the abundant surface hydroxyl groups and biological components; however, this is unlikely to be the sole source of adhesion, especially considering that similar hydrogen bonding sites could be produced on the surfaces of the tannic acid and hydrocaffeic acid coatings. The surface roughness at the nanoscale might be another key factor enhancing the cell adhesion. A rough surface with appropriately sized grooves or pores allows the anchoring of biological components, leading to cell residence^46^, and the surface texture even at the nanometre scale is known to enhance bacterial cell adhesion^47^. The non-toxic, nano-embossing of the modified surface contributed to the binding of cell compartments, resulting in enhanced cell viability. Furthermore, the homogeneity of the surface coating without uncovered areas probably enhanced the cell proliferation by eliminating local, microenvironmental restriction.

The adhesion of another cell line, PC12, was examined to verify the effects of DOPA-C7 modification further. The PC12 cell line is less adhesive than the NIH-3T3 cell line^21^, so PC12 adhesion could more clearly demonstrate the changes in adhesivity of the modified surface. As seen in the previous results, a large increase in cell adhesion was observed after the modification of the PTFE surface with DOPA-C7 (see SI, Fig. S8). In addition, patterning of PC12 cell adhesion on the DOPA-C7-modified surface is meaningful since the guided differentiation of embryonic PC12 cell into neuron-like cells is required in biomedical research. For example, in the field of regenerative medicine, PC12 cells have been used as a neural differentiation model^48^. In particular, recent studies have reported that the regulation of the direction of neural cell and axon growth is crucial for the functional recovery of nervous systems^49,50^. The DOPA-C7 assemblies demonstrated their potential as guides for the patterned differentiation of neural cells.

To investigate the effect of DOPA-C7 on the cell viability, we performed additional cell tests on the DOPA-C7 coated TCP surface. In these tests, TCP was chosen as the reference substrate to exclude the influence of cell adhesion; by observing the cell proliferation, the viability of the NIH-3T3 cell line on DOPA-C7 was clearly shown. The cell viability on the DOPA-C7 coated surface showed a modest enhancement from that of an untreated TCP, suggesting the excellent biocompatibility of DOPA-C7 and its negligible toxicity (see SI, Fig. S9). The enhanced cell adhesion and viability on the DOPA-C7-decorated TCP surface is indicative of the preferential interaction of the cellular components with the DOPA-C7 moieties. The textured surface and the high catechol density of the DOPA-C7 assembly presumably increased the stability of cell compartments on the surface and retarded protein denaturation at the interface between the cells and the heterogeneous surface^51,52^. The DOPA-C7 coating method allowed us to modulate the biointerface to regulate cellular events, including proliferation and adhesion, to a designed pattern.

**Figure 6:**
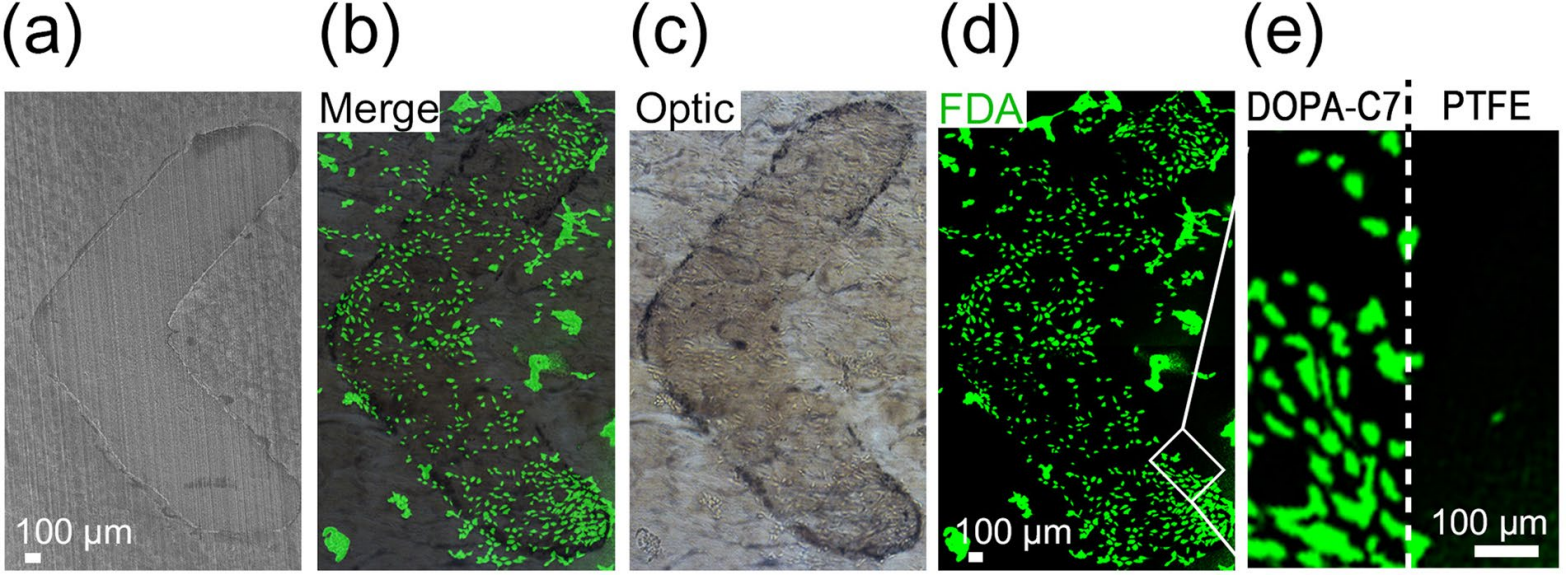
Adhesion and proliferation of NIH-3T3 cells on the patterned surface. (**a**) SEM image of the patterned PTFE surface that was prepared by inkjet printing using a DOPA-C7 suspension as the ink. (**b**–**d**) Optical and fluorescence microscopy images of the selective adhesion of NIH-3T3 cells on the patterned surface (scale bar: 100 μm). (**e**) Magnified fluorescence microscopy image at the border between the DOPA-C7 layer and PTFE.

**Figure 7:**
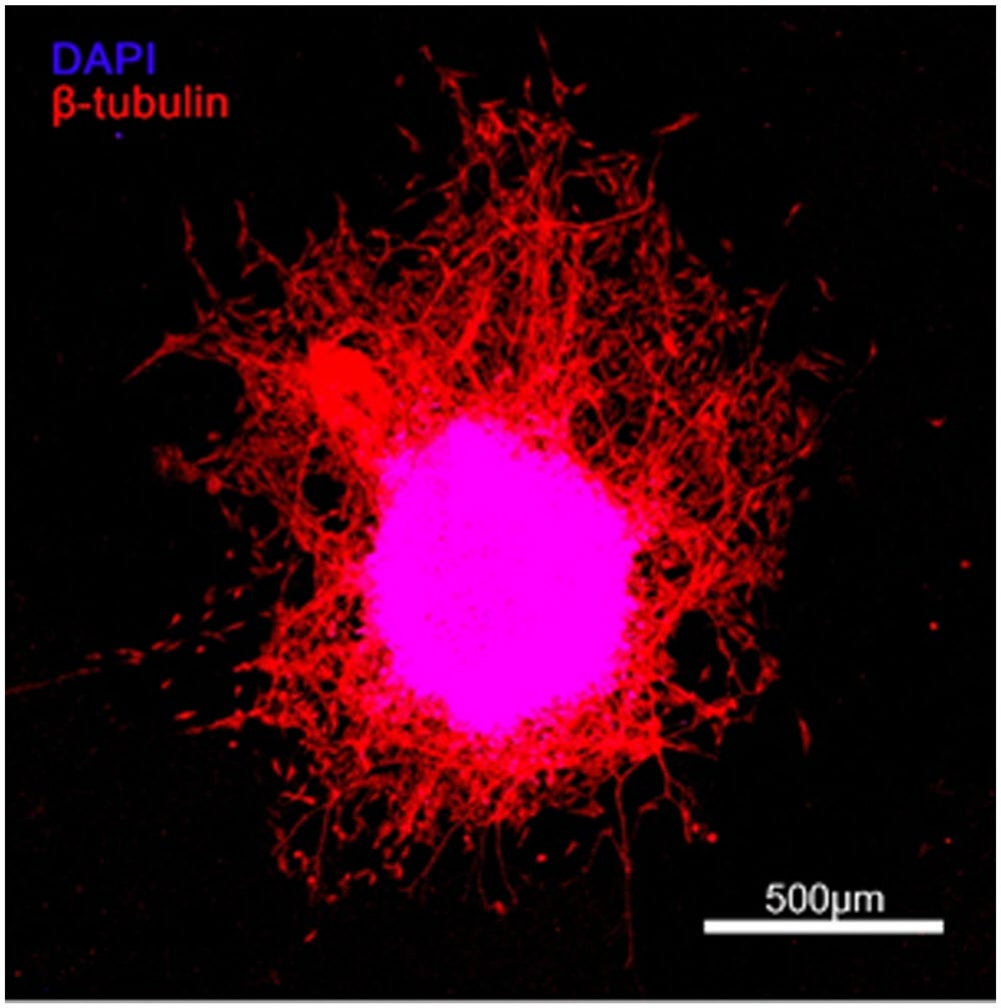
Neurite outgrowth from the dorsal root ganglion (DRG) on the DOPA-C7 coated PTFE substrate. The DRG was directly isolated from 9 days chick-embryos and cultured on the DOPA-C7 coated or non-modified PTFE surface under the NGF supplement (50 ng/mL). The differentiated neurites were detected by immunostaining with β-III tubulin antibody (red), and cellular nuclei were stained with DAPI (blue) at 2 days post-culture. The scale bar indicates 500 µm.

The prepared DOPA-C7 assembly solution was used as an ink and was printed on the PTFE film using a commercial inkjet printer. Inkjet printing is a facile and cheap method to prepare patterned surfaces. We prepared a simple chevron pattern, whose SEM image is shown in Fig. 6(a). The character “<” was 0.25 × 0.25 cm^2^ in size, and was prepared by five repeated printing of DOPA-C7. The optical microscopy image shows the deposition of nanoparticles on the PTFE surface (Fig. 6c), although the surface was not smooth because of the inherent roughness of the PTFE film. Both SEM and the optical images show that chevron patterning with a fine boundary was prepared. Spherical particulates were observed on the printed surface implying maintenance of the assembled structure after the inkjet printing (Fig. S10). The even distribution of NIH-3T3 cells without aggregation was observed mainly on the patterned surface (Fig. 6b), supporting the preferential adhesion of NIH-3T3 cells to the DOPA-C7 layer. This patterning method of inkjet printing with usage of the biochemically active, sticky assemblies is promising for the facile fabrication of biocompatible patterns on a variety of surfaces. Considering that only a small number of techniques, such as microcontact printing or lithography techniques, have been applied and the number of chemicals available for the creation of a cell-friendly patterned surface is limited, this versatile printing method, which does not result in the deterioration of the biomolecules, can be used in diverse applications. Importantly, as shown in Fig. 7, the surface modification with DOPA-C7 resulted in the stable adherence of primary cell clusters, dorsal root ganglia (DRG), which typically require the surface modification with adhesive agents, and led to robust neurite outgrowth under the supplement of nerve growth factor (NGF), further confirming the adhesive features of the DOPA-C7 treated surface. No primary cell clusters could be adhered on the unmodified PTFE surface.

## Conclusion

In summary, a solution of DOPA-bolaamphiphile assemblies was utilized as a non-toxic surface-modifying agent to add stickiness and nano-emboss to a variety of surfaces. Due to the stickiness of catechol and the spherical structure of the assemblies, the modified surfaces displayed enhanced cellular adhesion and viability. Many existing catechol-coating methods require two or more steps for the creation of sticky surfaces with complex surface structures^53,54^, however, our assembly-based method allows the preparation of sticky, complex structures in a single step and is applicable to patterning using a cheap inkjet printer. The results of surface modification and cellular adhesion indicate that the surfaces modified by DOPA-C7 could be used for cell patterning to mimic natural tissues. Therefore, this sticky assembly of DOPA-C7 molecules has potential for use in biomedical applications such as protein adsorption, cell adhesion, and biomolecule immobilization.

### Experimental Section

#### Chemicals

All chemicals were used as-received and without further purification, unless otherwise stated. For the synthesis of DOPA-C7, azelaic acid (98%, Aldrich) and 3,4-dihydroxyphenylalanine (DOPA, 98%, TLC) were used with the coupling reagents *N*-[3-(diethylamino)propyl]-*N*’-ethylcarbodiimide hydrochloride (EDAC, commercial grade, Aldrich) and *N*-hydroxysuccinimide (NHS, 98%, Aldrich). *N*,*N*-Dimethyl formamide (DMF, 99.5%), chloroform (extra pure grade), acetone (extra pure grade), and ethanol (extra pure grade) were purchased from Duksan Pure Chemical. The catechol compounds dopamine hydrochloride (98%), tannic acid (ACS reagent grade), hydrocaffeic acid (3,4-dihydroxyhydrocinnamic acid, 98%), and pyrocatechol (99%) were purchased from Sigma-Aldrich. Four substrates were examined for surface modification, including poly(tetrafluoroethylene) (PTFE; Hanmi Rubber & Plastics, Korea), glass (Marienfeld GmbH, Germany), polyethylene terephthalate (PET; DH3000, Dong Ho Company, Korea), and silicon rubber (Hanmi Rubber & Plastic, Korea).

NIH-3T3 (ATCC, CRL-1658) and PC12 cell lines (ATCC, CRL-1721) were used for the cell proliferation tests. For cell culture and counting, high-glucose Dulbecco’s Modification of Eagle’s Medium (high glucose DMEM; Corning Cellgro, USA), foetal bovine serum (FBS; Corning Cellgro, USA), penicillin-streptomycin (Pen/Strep; Thermo Fisher Scientific Inc., USA), a cell counting kit-8 (CCK-8; Dojindo Molecular Technologies Inc., Japan), 4′,6-diamidino-2-phenylindole (DAPI; Thermo Fisher Scientific Inc., USA), propidium iodide (PI; 94.0% HPLC, Sigma-Aldrich), and fluorescein diacetate (FDA, Sigma-Aldrich) were used as cell culture and counting agents.

#### Preparation of DOPA bolaamphiphile assemblies and surface modification

The DOPA-C7 bolaamphiphilic molecule was synthesized according to a previously reported protocol^28^. Briefly, the DOPA and azelaic acid were conjugated through the amide bond using the carbodiimide conjugation reaction. The synthesized DOPA-C7 molecules were collected and dissolved in tris-buffer solution (10 mM, pH 8.5) at a concentration of 5 mg/mL. In the solution, the DOPA-C7 molecules assembled to form nanospherical assembled structures, and this assembly solution was used as a surface-modifying solution. The substrates were rinsed in acetone and ethanol for 10 min to remove artefacts and dust. For the contact angle measurement, surface modification was achieved by immersing the substrate in the DOPA-C7 solution for 24 h. After 24 h, the solution turned light yellow, probably due to the oxidation of DOPA moieties, and the substrate was removed from the solution. The substrates were washed with deionized water and dried under a stream of nitrogen gas. For the pattern printing, the DOPA-C7 solution was transferred to an empty inkjet cartridge and was printed on a PTFE film using a commercial inkjet printer (Pixma ip2599, Canon).

#### Characterization

The surfaces of the modified substrates were observed using both field-emission scanning microscopy (FE-SEM, S-800, Hitachi, 20 kV) and AFM (Nanoscope III, Veeco). The chemical compositions of the surface before and after modification were examined by X-ray photoelectron spectroscopy (XPS, K-alpha, Thermo Scientific Inc., U.K.). Sessile-drop water contact angles were determined using a goniometer (Phoenix 300, Surface Electro-Optics Co., Korea). The adhesion strengths of DOPA-C7 and other catechol compounds (tannic acid, hydrocaffeic acid, and pyrocatechol) were determined from the pulling-off stress using a universal testing machine (UTM, MultiTest 1-i, Mecmesin). For the UTM tests, 5 μL solutions of the catechol compounds in tris-buffer (10 mM) were dropped and sandwiched between polyethylene terephthalate (PET) film strips in a contact area of 1 × 1 cm^2^ and were left at room temperature for 48 h to cure. The average failure stress of each adhesive compound was obtained after repeating the fracture test five times for each sample. Adhesion force versus distance curves were obtained by the force measurement method of AFM in contact mode (Nanoscope III, Veeco; Si_3_N_4_ cantilever: DNP-10, Veeco, spring constant of 0.2 N/m). The maximum adhesion force was recorded for every measurement and the average value was obtained from three measurements.

#### Cell Seeding

A 24-well tissue culture plate (TCP, Falcon, Corning Inc., USA) and PTFE substrates were coated with DOPA-C7 and other catechol materials. The surface-modified TCP and PTFE (0.5 cm × 0.5 cm) were transferred to a biosafety cabinet and UV sterilized for 12 h. After washing twice with a 1 × PBS solution, 500 μL of NIH-3T3 cells (4 × 10^4^ cells/mL) were seeded onto these modified substrates with cell culture media (1 × high-glucose DMEM, 10% FBS, 1% Pen/Strep). The cell-seeded plates were incubated at 37 °C in a 5% CO_2_ humidity chamber for 2 days.

#### Cell attachment and live/dead Assay

After seeding the NIH-3T3 cells for 2 days, the cell culture media was changed for 500 μL of fresh media. Then, the number of viable cells were quantitatively analysed using the cell counting kit-8 (CCK-8) assay. The CCK-8 assay was performed according to the CCK-8 kit protocol; briefly, 50 μL of CCK-8 dye was added to each well, and then the culture plate was incubated at 37 °C in a 5% CO_2_ humidity chamber for 3 h. The absorbance intensity (λ = 450 nm) of each modified surface was monitored using Nanodrop 2000 (Thermo Fisher Scientific Inc., USA) in UV-vis mode. Each experimental group was assayed individually in triplet, and all absorbance values were normalized by the average absorbance value of the conventional tissue culture plate.

The distribution of cell attachment and live/dead cells on each modified substrate was visualized by using 4,6-diamidino-2-phenylindole (DAPI) and fluorescein diacetate (FDA)/propidium iodide (PI) staining methods, respectively. After 48 h post-seeding, the conditioned media was removed in the 24-well plates and the cells were stained with the DAPI, FDA, and PI staining solutions composed of 3 μg/mL PI, 15 μg/mL FDA, and 15 μg/mL DAPI, respectively. After 30 min incubation at the 37 °C in a 5% CO_2_ humidity chamber, the cell-bound surfaces of the substrates were observed using fluorescence microscopy (Nikon, Japan)^55^. All experimental data were expressed as the average ± standard deviation. Statistical analyses were performed using a one-way analysis of variance (ANOVA) with the IBM SPSS 23.0 software (IBM Corporation, USA)

#### Dorsal Root Ganglion Culture

Dorsal root ganglia (DRG) were isolated from 9 days chick-embryos and cultured on the unmodified PTFE or DOPA-C7 coated PTFE substrates under the culture medium (DMEM/F12) supplemented with N2 (1%(v/v); Life Technologies) and nerve growth factor (NGF; 50 ng/mL, Prospec, Israel). At 3 days post-culture, the DRG cultured on each substrate were fixed by 4% paraformaldehyde (PFA) for 20 minutes at 4 °C and blocked by incubating with 5% goat serum (Sigma-Aldrich) in PBS-tween20 (PBS-T) for 2 hours at 25 °C. Neurites extended from the DRG were immunostained by mouse anti-β-III-tubulin primary antibody (1:500 dilution in PBS-T; Sigma-Aldrich) and goat anti-mouse Alexa 633 secondary antibody (1:250 dilution in PBS-T, Life Technology). The nuclei of DRG was stained by DAPI. Fluorescence images of DRG and neurite extension were acquired using a confocal laser microscope (CLSM, LSM 880, Carl Zeiss). All experimental procedures treating chick embryos were approved by Yonsei University Health System Institutional Animal Care and Use Committee. All experimental methods were carried out in accordance with the regulations and guidelines of Yonsei University.

## Electronic supplementary material

Supplementary Information
