# Supplementary material for: Enhanced Cell Adhesion on a Nano-Embossed, Sticky Surface Prepared by the Printing of a DOPA-Bolaamphiphile Assembly Ink
Source: Sci Rep. 2017 Oct 23;7:13797. doi: 10.1038/s41598-017-14249-4 (PMC5653752; doi:10.1038/s41598-017-14249-4)
Supplement: Supplementary file 1 — Supplementary Information [file 41598_2017_14249_MOESM1_ESM.pdf]

Supplementary Information

Enhanced Cell Adhesion on a Nano-Embossed, Sticky Surface Prepared by the Printing of a DOPA-Bolaamphiphile Assembly Ink

Chaemyeong Lee, Seung-Hyun Kim, Jae-Hyung Jang*, and Sang-Yup Lee*

Department of Chemical and Biomolecular Engineering, Yonsei University

50 Yonsei-ro, Seodaemun-gu, Seoul 120-749 KOREA

1. Surfaces coated with polydopamine (PDA) and DOPA-C7 assemblies

SEM images of the PDA-coated and DOPA-C7 assembly-coated silicon wafer surfaces are shown below.

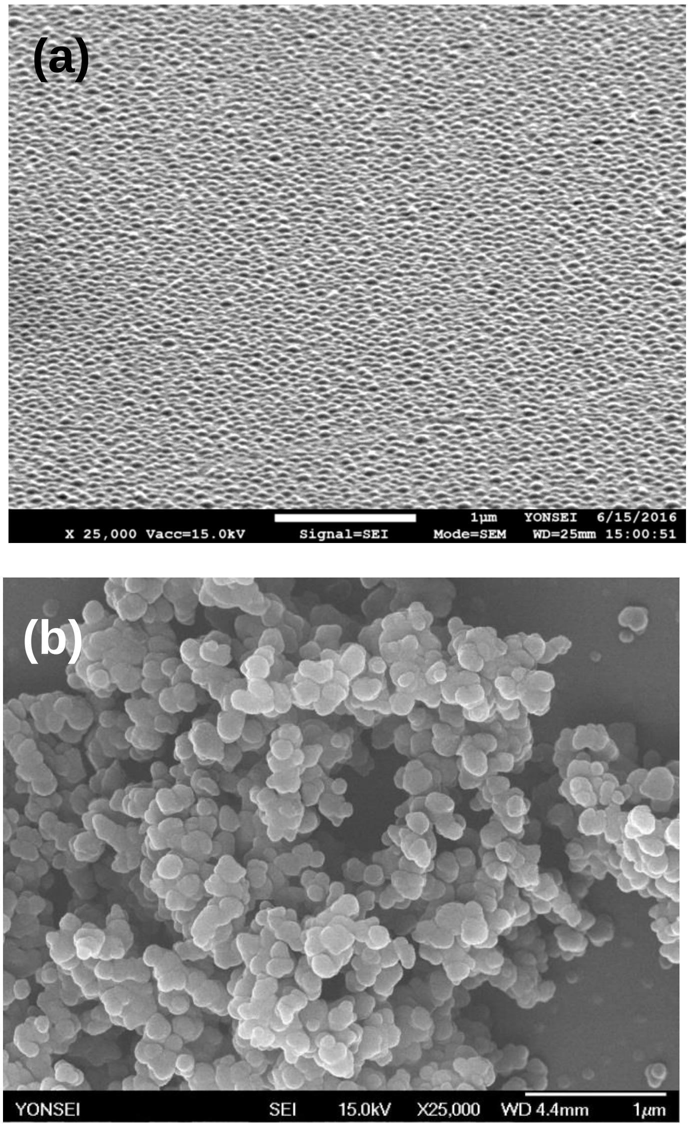

Figure S1. SEM images of silicon wafer surfaces coated with (a) DOPA-C7 assemblies (scale bar: 1 μm) and (b) polydopamine (scale bar: 1 μm).

2. Height profile of the DOPA-C7 assembly-coated surface

DOPA-C7 assemblies created nanoscaled bumps with ~ 10 nm depth. Height profile was determined from the topological image.

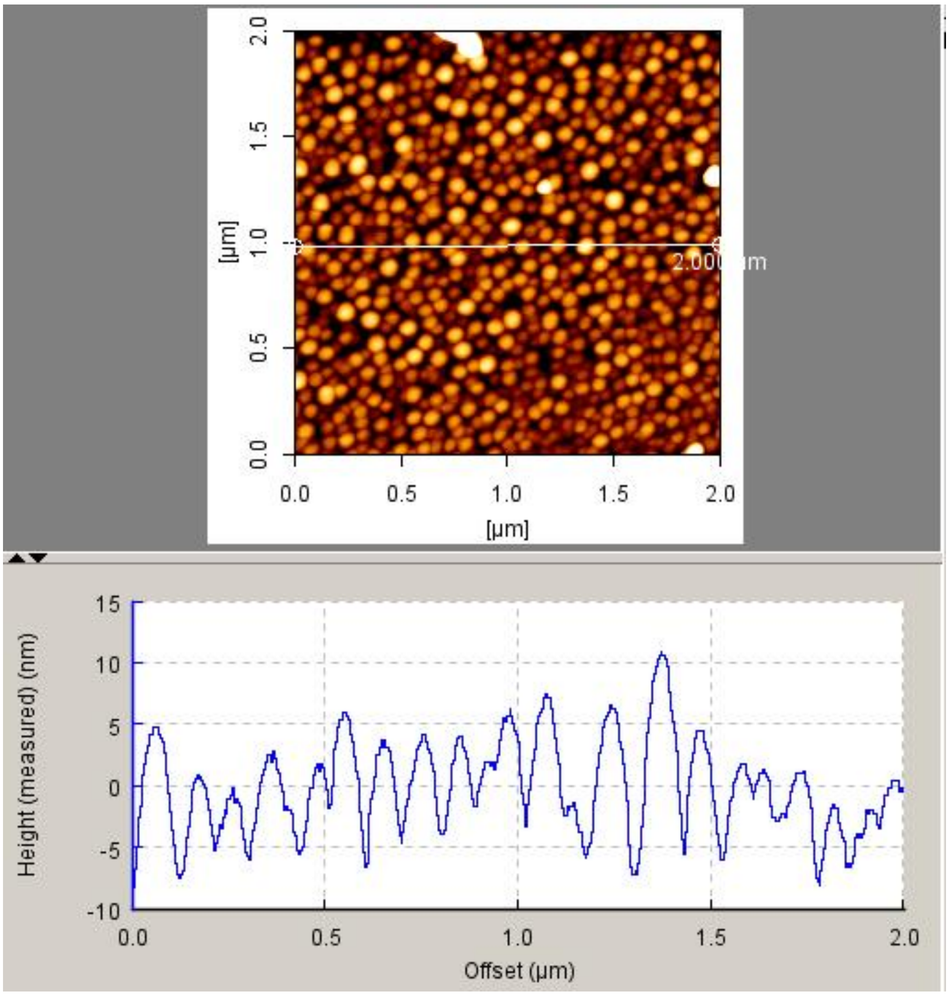

Figure S2. Topological image and corresponding height profile of DOPA-C7 assembly-decorated surface

3. Modification of various surfaces using DOPA-C7 assembly

To demonstrate the versatility of DOPA-C7 as a coating agent, various substrates were coated with DOPA-C7 assemblies and their water contact angles were measured. Water contact angle of hydrophobic substrates, such as PET and silicon rubber notably reduced after surface modification.

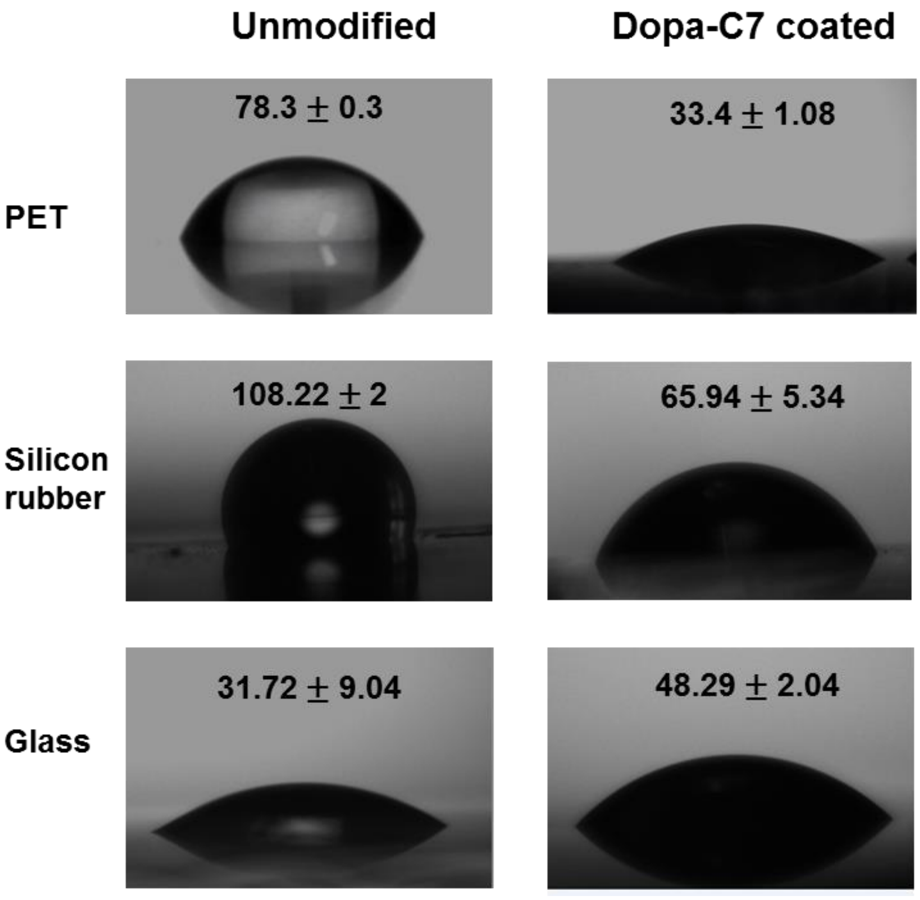

Figure S3. Changes of the water contact angles on various surfaces before/after deposition of DOPA-C7 assemblies.

4. Stress-strain curves of various catechol compounds

The adhesion property of DOPA-C7 and other catechol compounds were determined from the failure stress of the stress-strain curve. Elastic modulus was determined from the slope of the stress- strain curve.

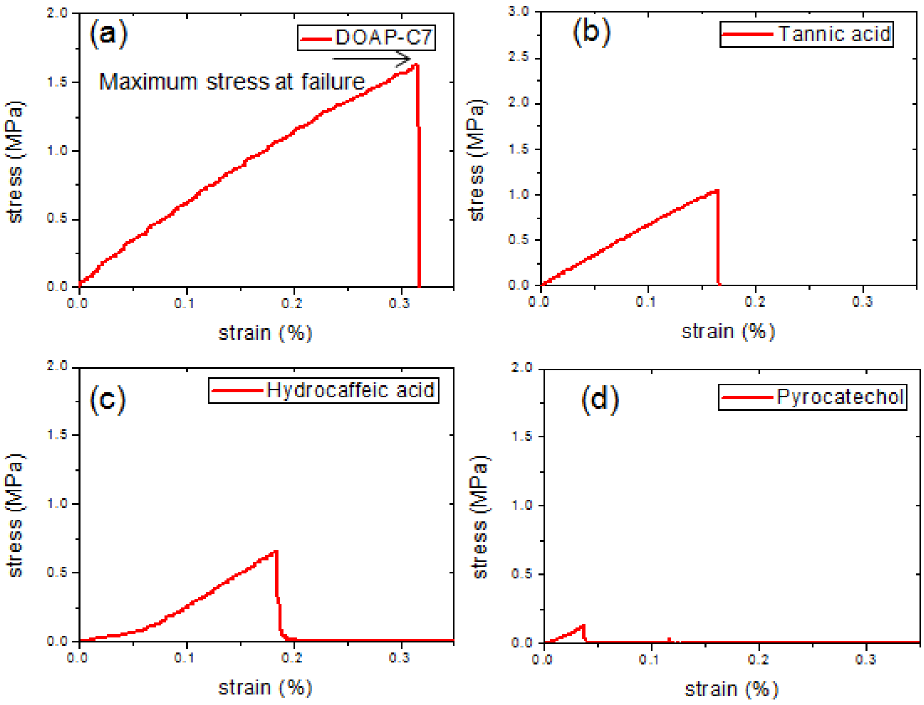

Figure S4. Stress-strain curve of each catechol compound. Adhesion strength was determined from the maximum stress at failure.

5. Fluorescence images of live and dead cells on each substrate

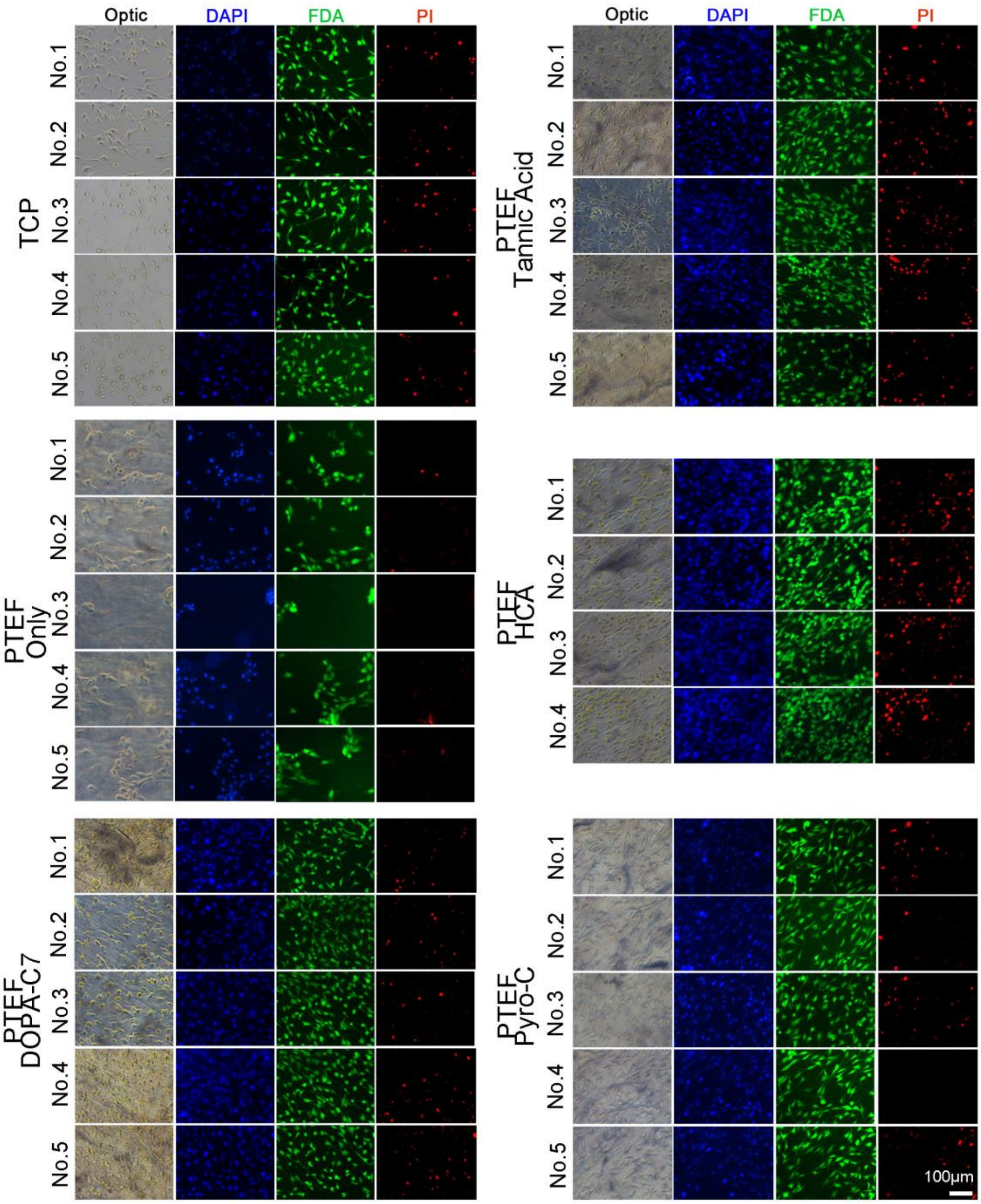

Figure S5. Optical and fluorescence microscopy images representing the DAPI-stained cellular nuclei (blue), FDA-stained live cells (green), and PI-stained dead cells (red) (scale bar: 100 μm). Representative fluorescence images were acquired from each condition with a lower magnification to visualize more cells on each substrate and shown in Fig. 5a.

6. Quantification of the number of live and dead cells

The FDA (live cells)- or PI (dead cells)-stained cells shown in Fig. S5 were manually counted, and the quantified numbers were related to the variations in cellular viability on each substrate.

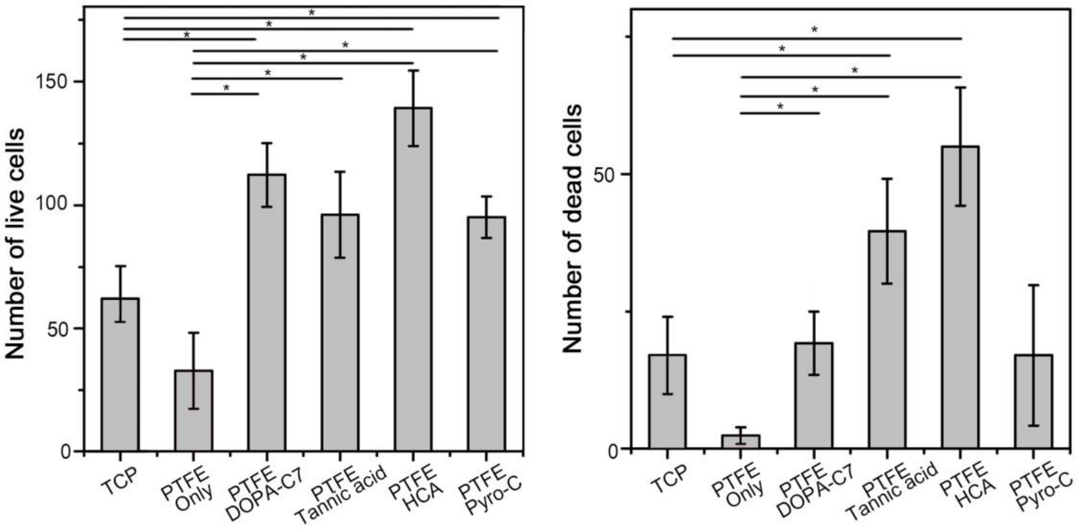

Figure S6. The number of live (left) and dead (right) cells on each surface. The symbol * indicates significant differences ($P<0.05$).

7. Cellular viability at 4 days of post-culture

The cellular proliferation was evaluated at 4 days post-culture by comparing the metabolic activities of the cells grown on each substrate with those on TCP. Cellular metabolic activities were assessed by using CCK-8 assay kit, and these results were shown in Figure S7.

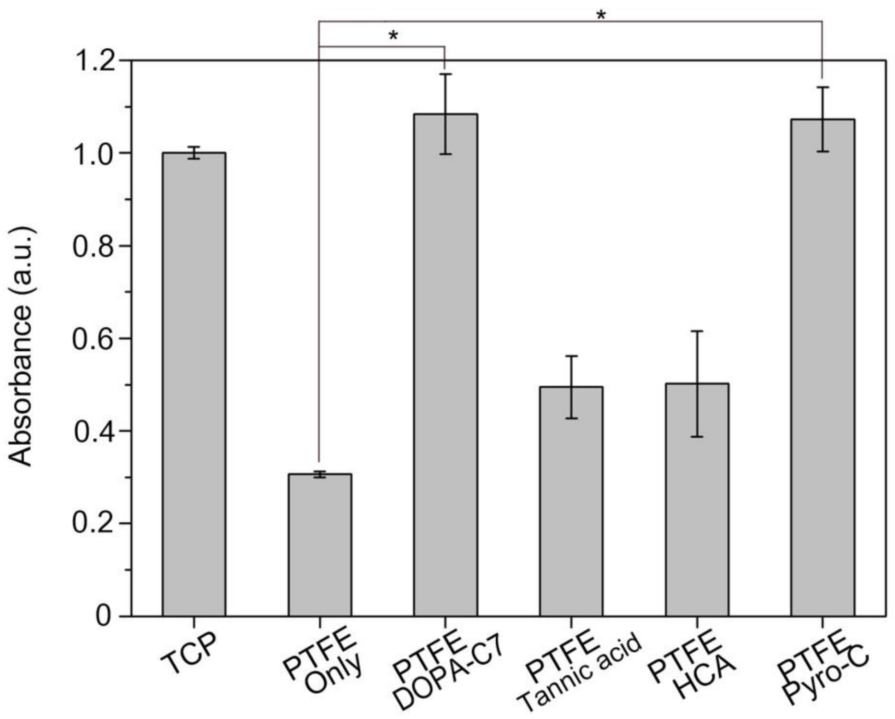

Figure S7. Cellular viability at 4 days post-culture

8. PC12 cell adhesion on the modified surface

Another cell line of PC12 was tested for the adhesion on the DOPA-C7 modified surface. PC12 is less adhesive to the commercial tissue culture plate (TCP) such that it can clearly display the effect of modified surface to improve cell adhesion. More PC12 cells were adhered on the DOPA-C7 modified surface even than TCP.

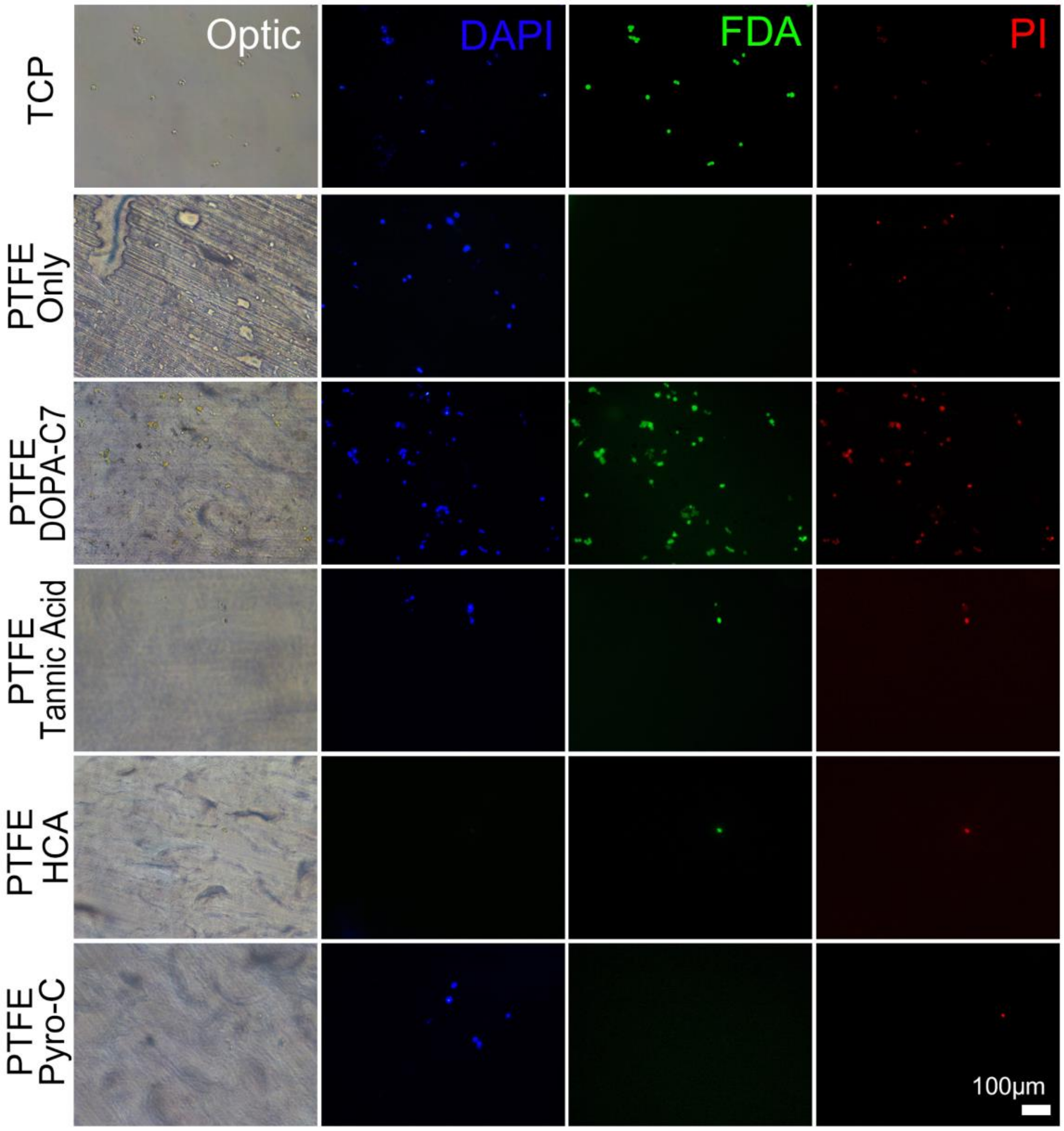

Figure S8. Optical and fluorescence microscopy images of PC12 cells adhered on the modified surfaces.

9. Cell viability test on DOPA-C7 coated TCP

Viability of NIH-3T3 cell on the DOPA-C7 coated TCP was examined. By using TCP as a substrate , the adhesion issue was excluded and the cell viability was focused only. Fluorescence image of the stained cells and CCK-8 assay indicate that there is little change in the cell viability when the modified TCP was used.

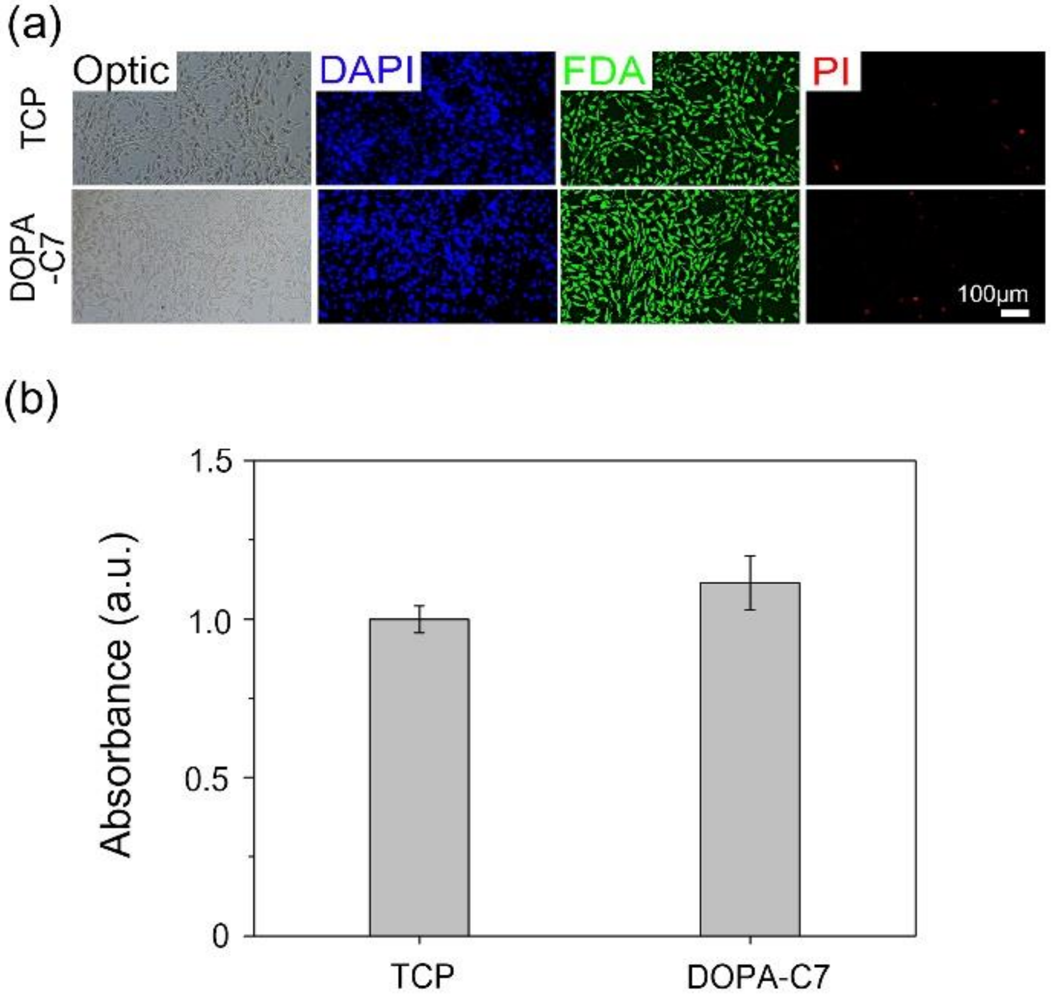

Figure S9. (a) Optical and fluorescence microscopy images of NIH-3T3 cells on the DOPA-C7 coated TCP, (b) Cell viability on TCP in the absence/presence of DOPA-C7 coating.

10. SEM image of the DOPA-C7 assemblies printed on the surface

SEM image of the PTFE surface printed with DOPA-C7 assemblies are shown in Figure S7. Intact PTFE surface was present for comparison. Spherical structure of the DOPA-C7 assemblies was remained intact even after the inkjet printing.

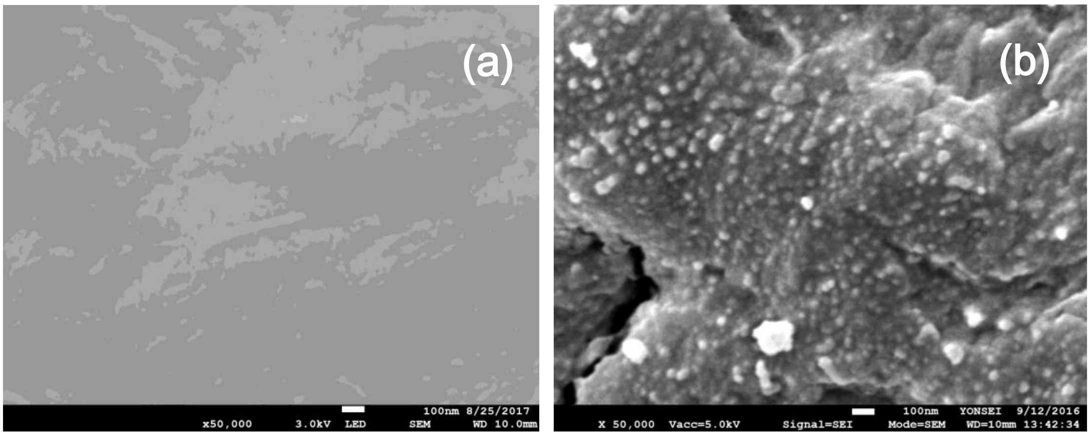

Figure S10. SEM image of the PTFE surface printed with DOPA-C7 assemblies. (a) Intact PTFE surface (scale bar: 100 nm), (b) PTFE surface deposited with DOPA-C7 assemblies (scale bar: 100 nm).
